# UPLC-PDA-ESI-QTOF-MS/MS fingerprint of purified flavonoid enriched fraction of *Bryophyllum pinnatum*; antioxidant properties, anticholinesterase activity and in *silico* studies

**DOI:** 10.1080/13880209.2021.1913189

**Published:** 2021-04-30

**Authors:** Joyce Oloaigbe Ogidigo, Chioma Assumpta Anosike, Parker Elijah Joshua, Collins U. Ibeji, Daniel Emmanuel Ekpo, Bennett C. Nwanguma, Okwesili Fred Chiletugo Nwodo

**Affiliations:** a Department of Biochemistry, Faculty of Biological Sciences, University of Nigeria, Nsukka, Nigeria; b Bioresources Development Centre, National Biotechnology Development Agency, Abuja, Nigeria; c Department of Pure and Industrial Chemistry, Faculty of Physical Sciences, University of Nigeria, Nsukka, Enugu State, Nigeria; d Department of Biochemistry, Mkar University, Benue State, Nigeria

**Keywords:** Alzheimer’s diseases, acetylcholinesterase, butyrylcholinesterase, carlinoside, docking studies, lipid peroxidation

## Abstract

**Context:**

*Bryophyllum pinnatum* (Lam.) Oken (Crassulaceae) is used traditionally to treat many ailments.

**Objectives:**

This study characterizes the constituents of *B. pinnatum* flavonoid-rich fraction (BPFRF) and investigates their antioxidant and anticholinesterase activity using *in vitro* and *in silico* approaches.

**Materials and methods:**

Methanol extract of *B. pinnatum* leaves was partitioned to yield the ethyl acetate fraction. BPFRF was isolated from the ethyl acetate fraction and purified. The constituent flavonoids were structurally characterized using UPLC-PDA-MS^2^. Antioxidant activity (DPPH), Fe^2+^-induced lipid peroxidation (LP) and anticholinesterase activity (Ellman’s method) of the BPFRF and standards (ascorbic acid and rivastigmine) across a concentration range of 3.125–100** **μg/mL were evaluated *in vitro* for 4 months. Molecular docking was performed to give insight into the binding potentials of BPFRF constituents against acetylcholinesterase (AChE) and butyrylcholinesterase (BuChE).

**Results:**

UPLC-PDA-MS^2^ analysis of BPFRF identified carlinoside, quercetin (most dominant), luteolin, isorhamnetin, luteolin-7-glucoside. Carlinoside was first reported in this plant. BPFRF significantly inhibited DPPH radical (IC_50_ = 7.382 ± 0.79 µg/mL) and LP (IC_50_ = 7.182 ± 0.60 µg/mL) better than quercetin and ascorbic acid. Also, BPFRF exhibited potent inhibition against AChE and BuChE with IC_50_ values of 22.283 ± 0.27 µg/mL and 33.437 ± 1.46 µg/mL, respectively compared to quercetin and rivastigmine. Docking studies revealed that luteolin-7-glucoside, carlinoside and quercetin interact effectively with crucial amino acid residues of AChE and BuChE through hydrogen bonds.

**Discussion and conclusions:**

BPFRF possesses an excellent natural source of cholinesterase inhibitor and antioxidant. The material could be further explored for the potential treatment of oxidative damage and cholinergic dysfunction in Alzheimer’s disease.

## Introduction

Alzheimer’s disease (AD) is a chronic, progressive age-related neurodegenerative disorder characterized by the manifestation of multiple cognitive impairments, gradual loss of memory and behavioural abilities (Vijayan and Reddy 2016). The pathophysiology of AD is complex and multiple aetiological factors such as the formation of senile plaques composed of amyloid-β (Aβ) protein (Swerdlow 2007; Qiu and Fratiglioni 2018), the formation of neurofibrillary tangles (NFT) (Wischik et al. 2014), oxidative stress and substantial loss of cholinergic function (Asaduzzaman et al. 2014) have been postulated to explain the progression of AD.

The enzyme cholinesterase is considered a major therapeutic target for the treatment of AD (Vinutha et al. 2007; Nwidu et al. 2017; Ali Reza et al. 2018; Moodie et al. 2019). Studies have reported a positive correlation between memory impairment in AD patients and a loss of cholinergic function as well as diminished levels of the neurotransmitter acetylcholine (ACh) in the brain (Asaduzzaman et al. 2014; Chlebek et al. 2019). Inhibition of acetylcholinesterase (AChE) and butyrylcholinesterase (BuChE) has been shown to increase neural acetylcholine levels and increase communication between nerve endings, which make them first-line therapy for alleviating symptomatic mild to moderate AD (Moodie et al. 2019). Several synthetic drugs currently used for symptomatic relief of AD such as donepezil, galantamine, and rivastigmine are found to slow the breakdown of acetylcholine and accordingly, reinforces cholinergic neurotransmission (Jiménez-González et al. 2018). However, these drugs have been marked with significant side effects and only show short-term relief from symptoms (Sun et al. 2012).

In addition to the role of cholinesterase enzyme in AD pathology, mounting experimental evidence has implicated oxidative damage in AD pathology resulting from β-amyloid deposition (Fischer and Maier 2015). Aβ has been found to initiate neuron death by several cellular reactions, such as the formation of reactive oxygen species (ROS) and reactive nitrogen species (RNS), oxidation of lipids, and protein (Chauhan and Chauhan 2006; Wu et al. 2016). Oxidative stress increased in an aging brain is caused by a disproportion of the redox state, involving the generation of excess ROS and resulting in the oxidation of biomolecules leading to cellular damage (Muller et al. 2007; Huang et al. 2016). Furthermore, the high utilization of oxygen by the central nervous system makes the brain tissues more susceptible to oxidative damage (Ademosun et al. 2016). Strong experimental evidence has shown elevated levels of lipid peroxidation products such as malondialdehyde (MDA) and hydroxynonenal in the brains of patients with AD (Huang et al. 2016). Antioxidant systems act to neutralize the possible destructive effects of ROS by the production of antioxidants to scavenge excess oxidants (Luca et al. 2015; Ali Reza et al. 2018).

*Bryophyllum pinnatum* (Lam.) Oken (Crassulaceae) is a succulent perennial herb commonly known as ‘miracle plant’ and in Nigeria locally known as ‘Oda opue’ in Igbo and ‘Abamoda’ in Yoruba. It is widely distributed in tropical Africa, India, China, Australia, and South America (Afzal et al. 2013). *Bryophyllum pinnatum* flourishes throughout parts of Southern Nigeria and is commonly used in folk medicine for the treatment of many ailments (Afzal et al. 2013; Chibli et al. 2014). Several bioactive compounds including, phenolics, alkaloids, flavonoids, triterpenes, glycosides, steroids, bufadienolides, cardenolides, and organic acids, have been identified and documented in *B. pinnatum* (Fernandes et al. 2019). *Bryophyllum pinnatum* plant extracts are reported to possess several pharmacological activities including antimicrobial (Abubakar et al. 2014), neuropharmacological (Salahdeen and Yemitan 2006), anti-inflammatory and analgesic (Fürer et al. 2013), muscle relaxant and sedative (Plangger et al. 2006), gastroprotective (Afzal et al. 2013) and antioxidant (Sharma et al. 2014).

Polyphenolic compounds such as flavonoids have gained increasing attention for their development as phytotherapeutics, owing to their roles in biological function such as antioxidant, antibacterial, anti-inflammatory, anticancer properties, their ability to delay age-related functional and physiological deficits in the brain, and their capacity to mediate important cellular enzyme functions (Panche et al. 2016; Bensalem et al. 2018). To date, there is still an untapped reservoir of substrates present in plant secondary metabolites that could serve as safer alternatives for the management of AD and other neurodegenerative diseases (Huang et al. 2016; Ali Reza et al. 2018; Khan, Ali, et al. 2018). A recent report by Ojo et al. (2018) and Elufioye et al. (2019) showed that *B. pinnatum* exhibits strong antioxidant and anticholinesterase activity. However, insights into the possible bioactive compound and mode of interaction are yet to be established. Hence, this study provides a detailed identification and characterization of the purified flavonoid enriched fraction of *B. pinnatum* leaves. For this purpose, a high-resolution ultra-performance liquid chromatography (UPLC) was applied for short-run times coupled with a photodiode array detector (PDA) and a quadrupole/time-of-flight mass spectrometer (Q/TOF-MS) and the antioxidant capacity and cholinesterase inhibitory activity were evaluated. Molecular docking studies were further employed to examine the possible molecular interactions of *B. pinnatum* flavonoid constituents against cholinesterase.

## Materials and methods

### Plant material procurement, identification and authentication

**Figure 1.**
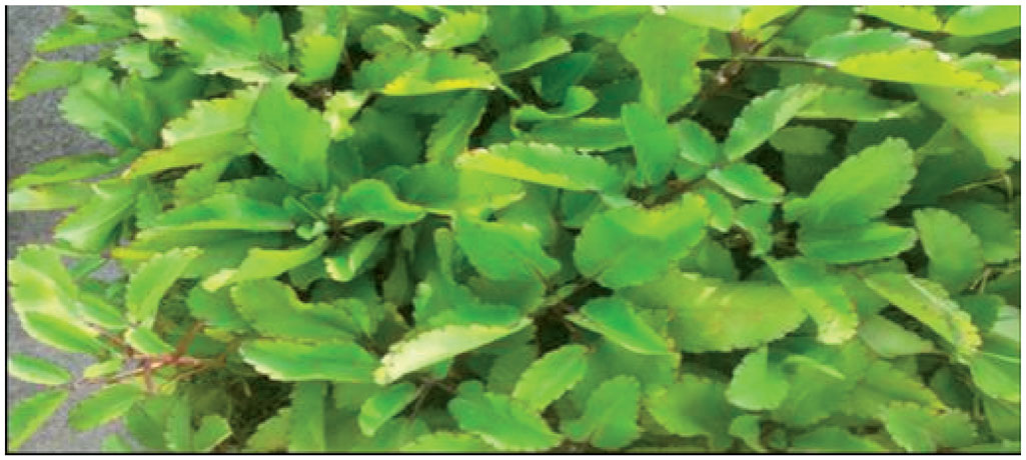
*Bryophyllum pinnatum* plant.

Fresh leaves of *B. pinnatum* **(**Figure 1**)** were collected around the Nsukka area of Enugu State, Nigeria, in June 2018. They were identified and authenticated by botanist Dr. Grace Ugbabe at the herbarium of the National Institute of Pharmaceutical Research Abuja, Nigeria. A voucher specimen was deposited, and a reference No: NIPRID/H/6855 was obtained.

### Plant preparation

The fresh leaves of *B. pinnatum* were carefully separated from the stem, washed with clean water to remove sand and debris, and then chopped into smaller pieces. The chopped leaves were spread under shade to drain completely. Subsequently, the leaves were air-dried to a constant weight. The dried plant leaves were pulverized into powdered form and stored in an air-tight container under room temperature (23–25 °C) devoid of light for two days.

### Chemicals and reagents

UHPLC-MS solvents, LC-MS formic acid reagent, acetonitrile were purchased from Merck (Santiago, Chile). Ultrapure water was obtained from a Millipore purification system (Milli-Q Merck Millipore, Santiago, Chile). UHPLC standards with a purity higher than 95% for HPLC) were acquired from Sigma Aldrich (St. Louis, MO, USA). Quercetin, ascorbic acid, gallic acid, sodium trioxocarbonate (VI), sodium phosphate, 1,1-diphenyl-2-picrylhydrazyl radical (DPPH), trichloroethanoic acid (TCA), thiobarbituric acid (TBA), ferrous sulphate, ethyl acetate, methanol, ethanol, Dowex^®^ 50WX8 hydrogen form, acetylcholine iodide (AChI), butyrylcholine iodide *(*BCEI) were purchased from Sigma Aldrich (St. Louis, MO, USA). Other reagents such as Folin-Ciocalteau, potassium bromide, hydrochloric acid, used for this study were of analytical grade and products procured from Qualikems Laboratories (India) and BDH Chemicals Ltd. (Poole, England). The water used for all analysis was glass distilled.

### Enzyme sources

Whole-brain tissue from adult male Wistar rat was used as a source of AChE enzyme and a lipid-rich media for lipid peroxidation assay while rat blood tissue was used as a source of BuChE enzyme. Animals were procured from the Department of Zoology, Faculty of Biological Sciences, University of Nigeria Nsukka. The animals used were kept in plastic cages and maintained in controlled conditions with a 12 h light/dark cycle at a temperature 23 ± 2 °C with access to food and water *ad libitium*. The protocols for animal handling were performed according to the National and International Ethical Recommendations for Care and Use of Laboratory Animals (National Academy of Science 2011) with the guidelines strictly observed. Ethical approval was obtained from the Ethics and Biosafety Committee, Faculty of Biological Sciences, UNN with protocol number UNN/FBS/EC/1009.

### Extraction of bioactive constituents

The preparation of the crude sample of *B. pinnatum* was carried out according to Kumar et al. (2014). A known weight of 1600 g of dried and grounded plant material was macerated with methanol (80%) in distilled deionized water and macerated for 72 h. The extract was filtered, and the filtrate was centrifuged to separate the fine suspended particles. Next, the filtrate was concentrated using a rotary evaporator to obtain crude (methanol) extract. The extract was suspended in water and partitioned in ethyl acetate to obtain ethyl acetate fraction which was used for ion-exchange chromatography.

### Resin activation and sample loading and elution

Activation of resin, isolation, and purification of flavonoid from the ethyl acetate fraction of *B. pinnatum* leaves as described previously by Omotuyi et al. (2018). Dowex 50WX8 (5 g) cation resin was activated by treatment with 2-bed volumes (BV) of ethanol overnight followed by rinsing with distilled water (5 BV) under room temperature (25 ± 1 °C). The activated resin was then loaded on a column and 200 mL of the fraction was loaded onto 50 mL resin volume in every cycle and allowed to settle for 1 h. The packed column was thoroughly washed with deionized water to elute unbound impurities and sugars. Bound flavonoids, also known as *B. pinnatum* flavonoid-rich fraction (BPFRF), were eluted using 5% HCL: 95% ethanol (1% v/v) until A_280_ nm was close to 0.000 and the eluted samples were collected and used for the assay.

### Phytochemical analysis

#### Estimation of total phenolic content (TPC)

The total phenolic content was measured following a described colorimetric method by Dohou et al. (2003). Gallic was used as standard and expressed as milligrams of gallic equivalent per gram of sample (mg GAE/g fraction). The total phenolic content was determined using the standard curve (*y* = 0.0066*x* + 0.0032; *R*^2^ = 0.9889) obtained with gallic acid.

#### Estimation of total flavonoid content (TFC)

The total flavonoid content was measured following a described colorimetric method (Zhishen et al. 1999). Quercetin was used as standard and expressed as milligrams of quercetin equivalent per gram of sample (mg QE/g sample). The flavonoid content was determined using the standard curve (*y* = 0.0052*x* + 0.0574; *R*^2^ = 0.9823) obtained with quercetin.

### *In vitro* bioactivity assay of BPFRF

#### *In vitro* antioxidant assay

##### 2,2-Diphenyl-1-picrylhydrazyl (DPPH) radical scavenging assay

DPPH free radical scavenging activity of BPFRF was determined using the DPPH spectrophotometric method of Brand-Williams et al. (1995) with slight modifications. Based on the principle, when DPPH reacts with an antioxidant compound that can donate hydrogen, it is reduced. The change in colour from deep violet to golden/light yellow can be measured at 518 nm. Briefly, 1 mL of 0.3 mM of DPPH solution was added to 1 mL of each of the test solutions. A BPFRF, quercetin or ascorbic acid (1 mL) at different concentrations (3.12–100 µg/mL) in 80% methanol was mixed with 0.5 mL of 0.076 mM DPPH in methanol. The mixtures were vortexed thoroughly and allowed to stand at room temperature in the dark for 30 min, and were incubated in the dark at room temperature for 30 min. The absorbance values were recorded at 518 nm, and converted into percentage antioxidant activity, using the formula below: Results were expressed as a percentage of inhibitions (100%). The concentration required to cause a 50% decrease in the absorbance (IC_50_) was, calculated from the standard plot. Each test was carried out in triplicates.

The % DPPH scavenging effect of the antioxidant was calculated as follows:

(1) $$\%\text{DPPH scavenging effect}=[\frac{A\text{ control }–\;A\;\mathrm{sample}}{A\mathrm{control}}]\times \;100$$

##### Fe^2+^-induced lipid peroxidation assay

Thiobarbituric acid reactive species (TBARS) assay was used to measure the lipid peroxide formed using brain tissue homogenates as lipid-rich media, as described by Ruberto et al. (2000) with slight modifications. Brain homogenate (10% v/v) (0.5 mL) was added to 0.1 mL of the BPFRF, quercetin or standard (ascorbic acid) (3.125–100 µg/mL) and the volume was then made up to 1.0 mL with distilled water. Thereafter, 0.05 mL of FeSO_4_ was added and the mixture was incubated at 37 °C for 30 min. Then, 1.5 mL of acetic acid was added, followed by 1.5 mL of TBA in SDS. The resulting mixture was mixed and heated at 95 °C for 60 min. After cooling, 5 mL of butanol was added and the mixture was centrifuged at 3000 rpm for 10 min. The absorbance of the organic upper layer was measured at 532 nm and the percentage inhibition was calculated with the formula:

(2) $$\%\text{inhibition}=[\frac{A\text{ control }–\;A\;\mathrm{sample}}{A\mathrm{control}}]\times 100$$

#### Acetylcholinesterase (AChE) and butyrylcholinesterase (BuChE) inhibitory activity

Ellman’s colorimetric method was applied to investigate the cholinesterase inhibitory activity of the BPFRF (Ellman et al. 1961). For the enzyme source, the rat brains were homogenized in a homogenizer with 10 volumes of a homogenization buffer (10 mM Tris-HCl, pH 7.2) containing 1 M NaCl, 50 mM MgCl_2_ and1% Triton X-100 and centrifuged at 10,000 rpm for 30 min. The resulting supernatants were collected, and the solution of these supernatants was used as an enzyme source. The temperature was maintained at 4 °C throughout the excretion procedure. The rate of AChE hydrolysis was monitored spectrophotometrically. Each BPFRF, quercetin or standard (rivastigmine) at varying concentrations (3.125–100 µg/mL) was mixed with an enzyme solution (500 μL) and incubated at 37 °C for 30 min. Absorbance at 405 nm was recorded immediately after adding Ellman’s reaction mixture (3.5 mL; 0.5 mM acetylthiocholine, 1 mM DTNB) in a 50 mM sodium phosphate buffer (pH 8.0) to the reaction mixture. Readings were repeated for 30 min to verify the reaction. Rivastigmine was used as a positive control. The percentage of AChE inhibition was calculated using the following formula:

(3) $$\%\text{Cholinesterase inhibition}=[\frac{A\text{ control }–\;A\;\mathrm{sample}}{A\mathrm{control}}]\times 100$$

Assessment of BuChE inhibition was performed as described in Equation (3) except that the enzyme solution was (100 μL) acetylthiocholine iodide was replaced with butyrylthiocholine iodide. All other reagents and conditions remained the same. The experiment was conducted in triplicate, and the concentrations of the test extract that inhibit the hydrolysis of the substrate (butyrylthiocholine) by 50% (IC_50_) were determined by linear regression analysis between the inhibition percentages against BPFRF concentration using Graph pad prism 5.0 program.

#### Identification and characterization of the flavonoid constituents in BPFRF

##### UPLC-PDA-ESI-QTOF-MS/MS instrumentation and chromatographic conditions

Qualitative and semi-quantitative UPLC-PDA-quadrupole time-of-flight mass spectrometer (MS) analysis of the BPFRF was carried out using the method of Stander et al. (2017). High-resolution UPLC-MS^2^ analysis was carried out using a Waters Synapt G2 quadrupole time-of-flight (QTOF) mass spectrometer (MS) coupled to an Acquity ultra-performance liquid chromatography (UPLC) (Waters, Milford, MA, USA). A Waters HSS T3, 2.1 × 100 mm, 1.7 μm column was used for successful separation and an injection volume of 2 μL was used. The mobile phase was made up of 0.1% formic acid (solvent A) and acetonitrile containing 0.1% formic acid as solvent B. The gradient started at 100% solvent A for 1 min and changed to 28% B over 22 min in a linear way. It then moved up to 40% B over 50 s and a wash step of 1.5 min at 100% B, followed by re-equilibration to initial conditions for 4 min. The flow rate was 0.3 mL/min and the column was maintained at a temperature of 55 °C. Ion mobility data were obtained using the same UPLC gradient and column as above and IMS Wave velocity was set at 332 m/s and wave height at 20.2 V. For the MS conditions, electrospray ionization was used in negative mode with a cone voltage of 15 V, desolvation temperature of 275 °C, desolvation gas at 650 L/h, and the rest of the MS settings optimized for best resolution and sensitivity. Data were obtained by scanning across a range of *m/z* 50 to 1500 in resolution mode as well as in MSE mode. In MSE mode, two channels of MS data were used, one at low collision energy (4 V) and the second using a collision energy ramp (40–100 V) to obtain fragmentation data as well. Leucine enkephalin was used as lock mass (reference mass) for accurate mass determination and the instrument was calibrated with sodium formate (Stander et al. 2017). Semi quantification of major flavonoids present in the BPFRF sample was evaluated from the peak areas in the UPLC profile using external calibration curves.

#### Molecular docking studies

The X-ray crystallographic structure of the cholinergic target proteins, that is, the human AChE and BuChE were downloaded from RCSB Protein Data Bank (PDB). 3D structures of AChE and BuChE with PDB ID: 4EY7 (resolution 2.3 Å) (Cheung et al. 2012) and 1POM (resolution 2.38 Å) (Nicolet et al. 2003), respectively, were used. Before molecular docking simulations, the two PDB protein structures were prepared via the ‘Protein Preparation Wizard’ workflow in the Schrodinger suit. Briefly, hydrogen atoms were added to the protein, bond orders were assigned, and unnecessary water molecules were deleted retaining only water molecules interacting with residues at the active site of the enzyme. Furthermore, side chains and missing residues were added, and the partial charges were assigned. Minimization of energy was carried out using OPLS_2005 (Optimised potentials for liquid simulations) force field. As all the downloaded proteins were co-crystallized structures, the ligand-binding site was used to define the active site of the protein. Receptor grid generation workflow was used to define a grid (box) around the ligand, to keep all the functional residues in the grid (Sastry et al. 2013; Kumar et al. 2017). 2D structures of the identified ligands obtained from UPLC-PDA-ESI-MS/MS data were retrieved from the PubChem database. Ligprep was used to prepare and optimize the ligands prior to docking (Friesner et al. 2006); extra precision (XP) docking was employed to dock the prepared protein and the ligands. Structures of ligands were set as flexible to generate several conformations. Calculations were performed using the OPLS_2005 force field (Kumar et al. 2017). All the results were analyzed in XP visualizer.

### Statistical analysis

The data obtained from the study were analyzed using Graph pad prism version 5.0. The results were expressed as mean ± standard deviation of triplicate measurements.

## Results and discussion

### Percentage yield of BPFRF

The extraction of 1.6 kg of the powdered leaves of *B. pinnatum* with methanol gave a yield of 4.77% of the powdered plant material soaked. Partitioning of 77 g of the MEBP partitioned in ethyl acetate resulted in 7.69 g of the ethyl acetate partition (EAF). Subsequent purification of the EAF by ion-exchange chromatography led to the formation of a purified flavonoid-rich fraction (BPFRF) with a percentage yield of 9.97%. The high BPFRF yield observed could be attributed to the purity of the fraction and method of purification (Omotuyi et al. 2018). Extraction solvent, nature, and conditions of the leaves, acidification percentage, drying temperature and storage conditions are important factors to be considered for the optimal yield of flavonoids from plants (John et al. 2005).

### Total phenolic and flavonoid concentrations of the BPFRF

*Bryophyllum pinnatum* flavonoid-rich fraction (BPFRF) was examined for its total flavonoid and phenolic contents. The results indicated that the total flavonoid content of BPFRF was 132.87 ± 0.10 mg QE/g while the total phenolic contents were 83.98 ± 0.37 mg GAE/g. This result is incongruent with previously reported literature carried out on the methanol extract and ethyl acetate fraction of *B. pinnatum* leaves (Ojo et al. 2018). The enriched flavonoid content observed in this study could be attributed to the purification process; this is in agreement with the previous study by Liang et al. (2019). Polyphenols have been found to possess high antioxidant activity as well as a plethora of favourable benefits to human health (Queen and Tollefsbol 2010; Ben Haj Yahia et al. 2019).

### Identification and characterization of BPFRF by UPLC-ESI-QTOF-MS^2^

**Figure 2.**
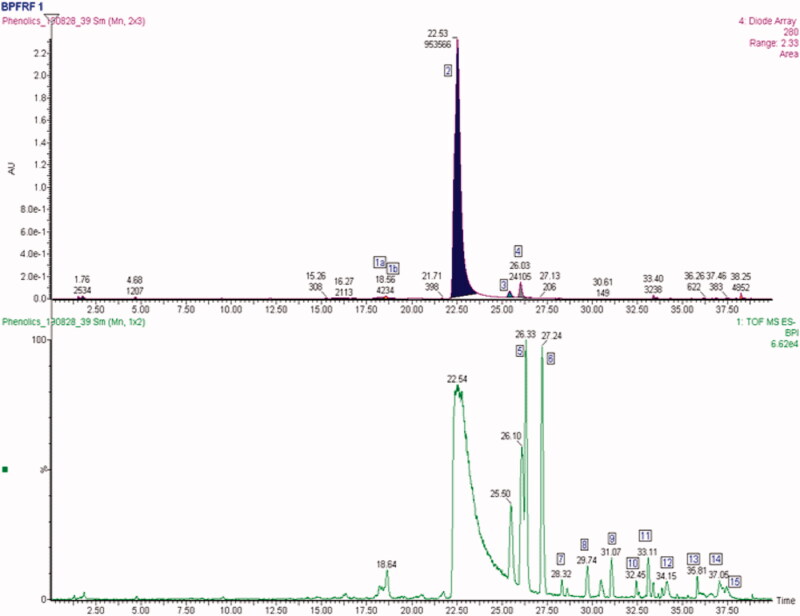
Overlay of UPLC chromatogram at 280** **nm top and base peak intensity (BPI) chromatograms (bottom) of BPFRF.

**Figure 3.**
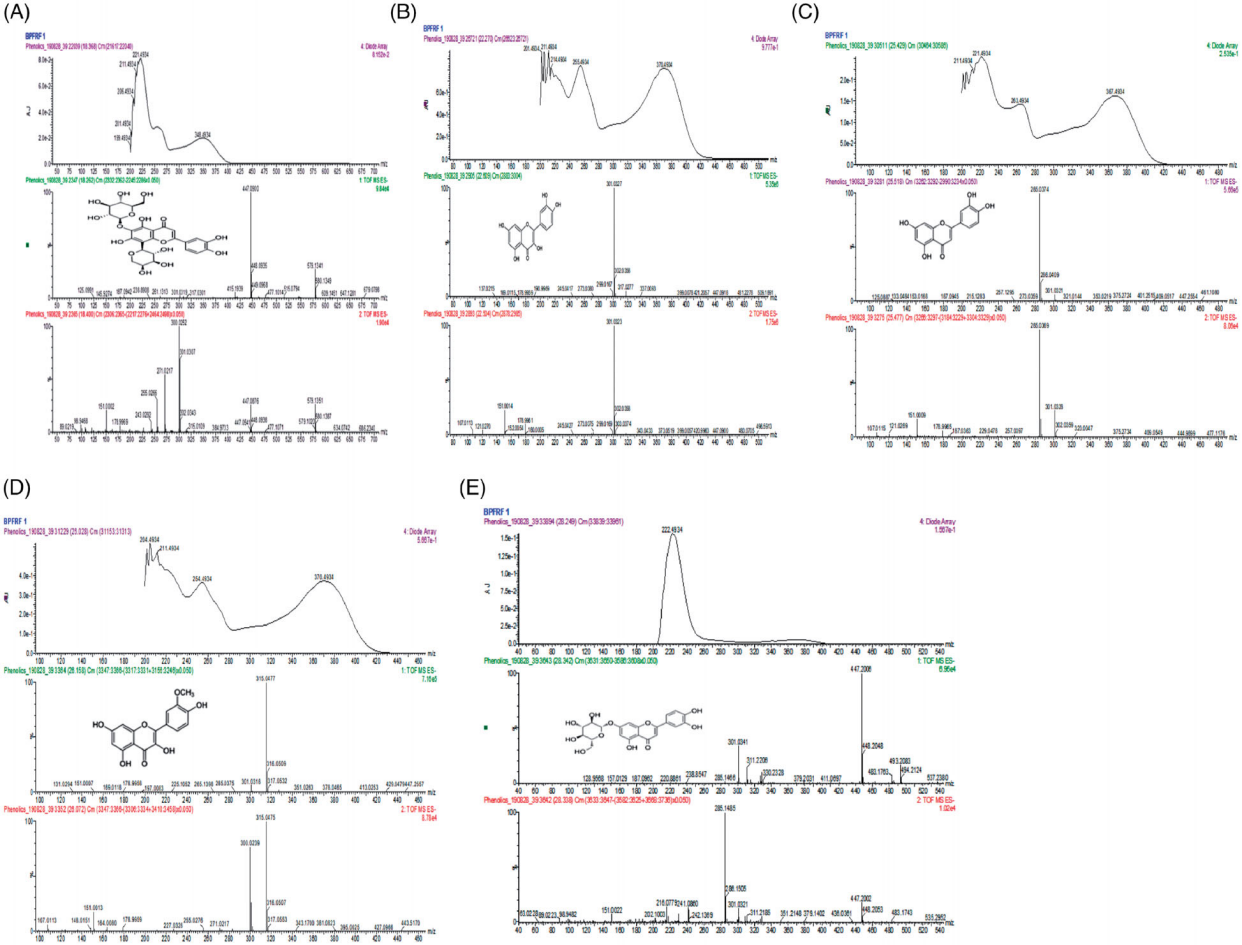
Photodiode array detector (PDA) spectra and mass spectra (MS/MS) fragmentation pattern present in *B. pinnatum* flavonoid-rich fraction (BPFRF). Luteolin C-glucoside-C-arabinoside (carlinoside) (A). Quercetin (B). Luteolin (C). Quercetin-3-methyl ether (isorhamnetin) (D). Luteolin-7-glucoside (E).

Several analytical techniques such as UV spectrometry, HPLC-DAD, GC-MS, HPLC-MS/MS, have been applied for the identification of polyphenolic compounds (Yang et al. 2020). UPLC-PDA-ESI-QTOF-MS^2^ has been reported to be most effective and sensitive for accurately identifying and characterizing the structures of known and unknown compounds (Yang et al. 2020). In this study, UPLC-ESI-QTOF-MS^2^ technique was employed in profiling and characterizing the flavonoid enriched fraction of *B. pinnatum* (BPFRF). Structures of BPFRF chemical compounds were detected and characterized by comparing their chromatographic and spectrometric data with scientific literature, open-access mass-spectra databases and authentic standards were available. The UV chromatogram and PDA spectra showed several peaks of UV-absorption of BPFRF compounds. Also, a cluster of large peaks was observed between 16 and 27 min. The UPLC-PDA-QTOF-ESI-MS/MS patterns of BPFRF are summarised in Table 1 and Figure 2, respectively. Peak **1a** (compound **1a**) was eluted at (Rt) 18.56 min (Table 1 and Figure 2) and exhibited UV spectra detected at (λ max of 255 and 370 nm) (Figure 3(A)). Compound **1a** exhibited a molecular ion [M–H]^–^ at *m/z* 579.1341 and daughter fragments at *m/z* 447, 415, 300, 301, 255, 243, 217, 178 (Figure 3(A)). The resulting elemental composition of compound **1a** was (C_26_H_27_O_15_). So, compound **1a** was tentatively identified as carlinoside, a flavone derivative of luteolin (8-arabinopyranosyl-6-glucopyranosyl luteolin). This compound’s mass spectra data is consistent with a similar compound present in Rooibos tea and reported by Stander et al. (2017). Peak (**2**) eluted at retention time 22.51 min (Table 1 and Figure 2) and indicated a characteristic UV spectrum at 254 nm and 370 nm (Figure 3(B)). The [M–H]^−^ ion parent fragment was *m/z* 301.0327 with molecular formula C_15_H_9_O_7_. The daughter fragmentation patterns generated were *m/z* 299, 273, 245, 178, 152, 121, 107 of different compounds fragmented from the parent flavonoid identified (Figure 3(B)). The identification of compound **2** was confirmed by comparing its UPLC retention time, UV and mass spectra which was similar to those reported in previous literature (Brito et al. 2014; Oufir et al. 2015; Garayev et al. 2018) quercetin (compound **2**) was found to be the most abundant flavonoid in BPFRF sample with an estimated peak area of about 80%. Peak (**3**) eluted at (Rt) 25.50 min (Table 1 and Figure 2) and showed UV maxima at 263 nm and 367 nm (Figure 3(C)) which is characteristic of flavones showing parent ions of *m/z* 285.0374 [M–H]^−^ and assigned molecular formula of C_15_H_9_O_6_. The fragmentation patterns of *m/z* 301, 257, 229, 187, 178, 151, 121, 107 (Figure 3(C)), showing that compound **3** is an aglycone flavone identified as luteolin. Similar fragmentation patterns were observed from the parent compound previously reported by Li et al. (2018) and Stander et al. (2017). Peak **4** was eluted at the retention time of 26.03 min (Table 1 and Figure 2) with UV maxima of 254 nm and 370 nm (Figure 3(D)) which is characteristic of flavonols showing parent ions of *m/z* 315.0477 [M–H]^−^ and presents a molecular formula of C_16_H_11_O_7_. The mass fragmentation patterns with intensity normalized to 80 for the highest fragment resulted in a quercetin fragment at 300 (80), and other daughter fragment ions of *m/z* 300, 271, 255, 227, 178, 151, 148, 107 (Figure 3(D)), indicating that compound **4** is an aglycone flavonol identified as isorhamnetin (quercetin-3-methyl ether) (Garayev et al. 2018). Peak **7** was eluted at the retention time of 28.32 min (Table 1 and Figure 2) with UV maxima of 222.41 nm and 367 nm (Figure 3(E)), which is characteristic of flavones showing parent ions of *m/z* 447.2006 [M–H]^−^ and presents a molecular formula of C_21_H_20_O_11_. The mass fragmentation patterns with intensity normalized to 100 for the highest fragment resulted in a luteolin fragment at 285.14 (100), and other daughter fragment ions of *m/z* 351, 311, 301, 286, 241, 216, 202,151 (Figure 3(E)), showing that compound **7** is an aglycone flavone luteolin-7-glycoside (cynaroside) (Li et al. 2018). To the best of our knowledge, carlinoside (8-arabinopyranosyl-6-glucopyranosyl luteolin) had not been previously reported as a constituent of *B. pinnatum*, while compounds **2–4** were already described (Fürer et al. 2013). It is noteworthy that of the 15 compounds detected and separated by the UPLC only the 5 flavonoid compounds were tentatively identified which mainly belong to two principal metabolite types: flavone and flavonol class of flavonoids. These flavonoids have been reported to possess potent anticholinesterase activity (Panche et al. 2016; Khan, Marya, et al. 2018) and neuroprotective effects (Khan, Ali, et al. 2018).

**Table 1.** Constituents of BPFRF identified and characterized by UPLC-PDA-Q/TOF-MS^2^ analysis.

Peaks	**Retention time** **(min)**	**UPLC PDA** **λmax** **(nm)**	^a^m/z	^b^M-H^−^	^c^MS^E^(MS/MS)	**Concentration** **g/100 g**	**Tentative** **Identification**
**1a**	18.56	221, 348	579.1341	C_26_H_27_O_15_	151, 178,243, 255,271, 300,315,447	0.27	**8-Arabinopyranosyl-6-glucopyranosyl luteolin (carlinoside)**
**2**	22.54	255, 370	301.0327	C_15_H_9_O_7_	107,151,178,190,245,273	13.34	**Quercetin**
**3**	25.50	263, 367	285.0374	C_15_H_9_O_6_	107,121,151,178,187,229,257	2.01	**Luteolin**
**4**	26.03	254,370	315.0477	C_16_H_11_O_7_	107,148,151,164,178,227,255,277	3.49	**Quercetin methyl ester (isorhamnetin)**
**5**	26.33	–	327.2107	C_18_H_31_O_5_	127,151,171,183,211,229,239,291, 300, 301,315	**-**	**Unknown**
**6**	27.24	221,367	329.1502	C_18_H_33_O_5_	127,151,171,183,211,229,233,251,300, 301	**-**	**Unknown**
**7**	28.32	222.41	447.2006	C_24_H_31_O_8_	89, 151,202, 216, 241, 285, 301, 311, 351, 379	0.92	**luteolin-7-glucoside**
**8**	29.74	223.49	795.4507	C_42_H_67_O_14_	119,183,236,301,339, 457, 507, 615, 647, 691, 765	**-**	**Unknown**
**9**	31.07	No UV	633.3901	C_36_H_57_O_9_	85, 113, 116,151, 183, 220, 301, 339, 407, 463, 485, 559	**-**	**Unknown**
**10**	32.45	No UV	293.2134	C_18_H_29_O_6_	98, 116, 119, 121, 169, 171, 183, 235, 236,275	**-**	**Unknown**
**11**	33.11	No UV	617.4011	C_36_H_57_O_8_	85, 113, 151, 235, 253, 277, 339, 367,439, 497, 513, 579	**-**	**Unknown**
**12**	34.15	226	265.1402	C_15_H_21_O_4_	98, 116, 151, 183, 220, 255, 279	**-**	**Unknown**
**13**	35.81		271.2201	C_13_H_27_O_8_	130, 150, 171, 183, 197,221, 220, 226, 255, 235	**-**	**Unknown**
**14**	37.05	226	271.2214	C_13_H_27_O_8_	96, 98, 100, 119, 139, 183, 199, 255, 265, 293, 325, 353, 397, 465	**-**	**Unknown**
**15**	37.05	226	325.1812	C_14_H_29_O_8_	79, 96, 119, 170, 183, 184, 197, 227, 265,293	**-**	**Unknown**

^a^Accurate mass detection; ^b^negative ions; ^c^mass fragmentation patterns with intensity normalised to 100 for the highest fragment. Rt: Retention time; λmax: maxima absorbance.

### *In vitro* bioactivity assay of BPFRF

#### DPPH radical scavenging activity

**Figure 4.**
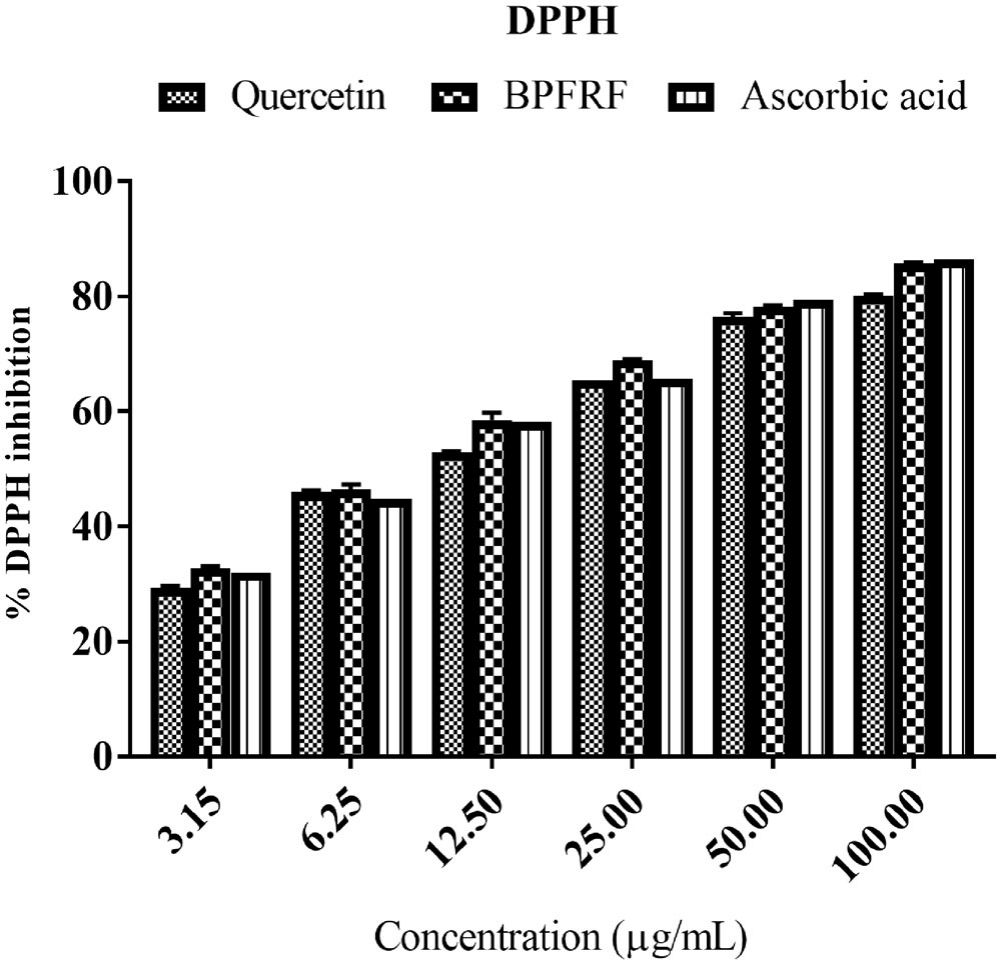
2,2-Diphenyl-1-picrylhydrazyl free radical scavenging effect of BPFRF in comparison with ascorbic acid (3.125–100** **μg/mL).

Evaluation of the DPPH radical scavenging property of the BPFRF is presented in Figure 4 and Table 2. From our result, BPFRF, quercetin, and ascorbic acid caused a concentration-dependent increase in the inhibition of the DPPH radical Figure 4. DPPH is a stable nitrogen-based free radical that functions as a substrate of radical-trapping reactions in this method (Assefa et al. 2016; Ben Haj Yahia et al. 2019). The BPFRF caused a fast conversion of the purple-colored DPPH radical to pale yellow. Reduction in absorbance shows the antioxidant activity of the fraction (Assefa et al. 2016). As shown in Table. 2, BPFRF was found to be a better DPPH radical scavenger with an estimated IC_50_ value of 7.382** **±** **0.79** **µg/mL and *R*^2^ = 0.731 than quercetin (IC_50_ = 13.803** **±** **0.34** **µg/mL and *R*^2^ = 0.741) and the reference standard ascorbic (IC_50_ = 10.186** **±** **0.14** **µg/mL and *R*^2^ = 0.76). The IC_50_ values in µM were also presented for comparison, See Supplementary Table 1. This result agrees with previous studies that showed the radical scavenging activity of *B. pinnatium* crude extract against DPPH radical (Ojo et al. 2018; Gupta et al. 2016). Moreover, UPLC-PDA-QTOF-MS/MS identification of BPFRF showed the presence of some flavonoids (Table 1) (i.e., carlinoside, quercetin (most abundant), luteolin, isorhamnetin and luteolin-7-glucoside) with evident antioxidant ability. These flavonoids may be mostly responsible for the potent antioxidant abilities exhibited by BPFRF. Quercetin, luteolin, isorhamnetin are potent radical scavengers owing to their ability to reduce the stable DPPH radical either by their capacity to donate a hydrogen atom or electron; thus, further converting the radicals into non-toxic species (Rahman et al. 2015).

**Table 2.** DPPH radical scavenging potential, lipid peroxidation inhibitory activity and cholinesterase inhibitory activity of BPFRF, quercetin and standards.

Samples	DPPH radical IC_50_ (µg/mL)	Lipid peroxidation IC_50_ (µg/mL)	AChE IC_50_ (µg/mL)	BuChE IC_50_ (µg/mL)
BPFRF	7.382 ± 0.79	7.182 ± 0.60	22.283 ± 0.27	33.437 ± 1.46
Ascorbic acid	10.186 ± 0.14	14.393 ± 0.54		
Quercetin	13.803 ± 0.34	25.242 ± 1.97	15.528 ± 0.08	24.326 ± 0.40
Rivastigamine			7.994 ± 0.12	20.120 ± 0.72

#### Lipid peroxidation inhibition activity

**Figure 5.**
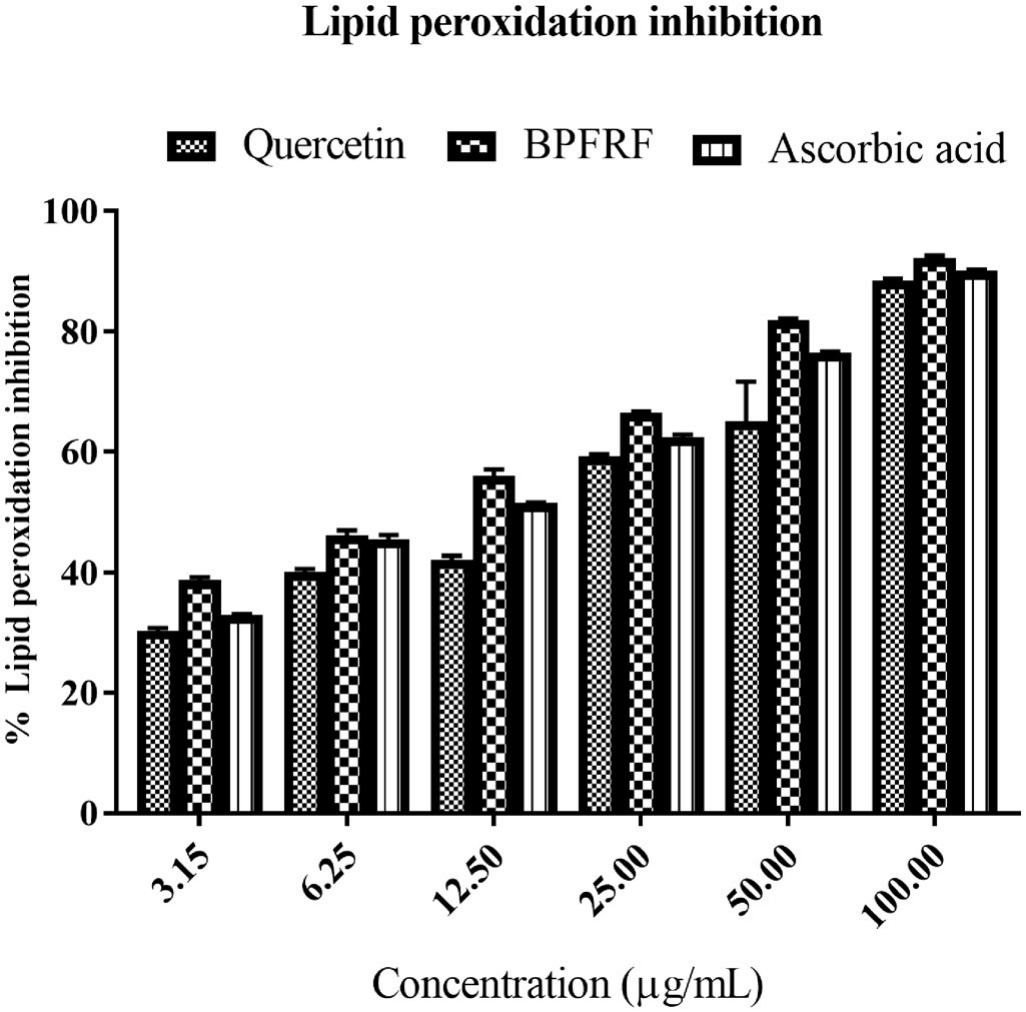
Fe^2+^-induced lipid peroxidation inhibitory activity of BPFRF and the ascorbic acid.

This study evaluated the inhibitory effect of BPFRF against non-enzymatic lipid peroxidation in rat brain homogenate. The inhibition of lipid peroxidation is considered the most important marker of oxidative stress (Badmus et al. 2011). *In vitro* lipid peroxidation was initiated by incubating rat brain tissue in the presence of FeSO_4_. Our results revealed that BPFRF, quercetin and the standard (ascorbic acid) inhibited Fe^2+^ induced lipid peroxidation in a dose-dependent manner Figure 5. The IC_50_ (fraction concentration causing 50% inhibition of LP) values are presented in Table 2, BPFRF (IC_50_ = 7.182** **±** **0.60** **µg/mL and *R*^2^ = 0.86 had a higher inhibitory effect on Fe^2+^ induced lipid peroxidation in the brain homogenate compared to standard ascorbic acid (IC_50_ = 14.393** **±** **0.54** **µg/mL and *R*^2^ = 0.880) and quercetin (IC_50_ = 25.242** **±** **1.97** **µg/mL and *R*^2^ = 0.92). The IC_50_ values in µM were also presented for comparison, See Supplementary Table 1. These results are in agreement with the previous study carried out by Gupta et al. (2016). These results showed that BPFRF can prevent cellular aberrations caused by ROS by breaking down the chain reactions responsible for lipid peroxidation (Ali Reza et al. 2018). The result obtained from the present study suggests the peroxide trapping property of BPFRF constituents. Brain tissues are made up of lipid-rich medium normally susceptible to per-oxidative attack (Chauhan and Chauhan 2006). The process of lipid oxidation is preceded by hydrogen atom removal from an unsaturated fatty acid, culminating in lipid peroxyl radical formation (Tönnies and Trushina 2017). Thus, the ability of BPFRF to inhibit Fe^2+^ mediated lipid peroxidation may largely be attributed to their distinct chemical structures, such as the number and position of hydroxyls and presence of double bonds on aromatic rings A and B as well as the heterocyclic ring C of the flavonoids identified in BPFRF (Table 1) which donate H atom to fatty acyl chains in the membranes and scavenge OH radicals generated by Fenton’s reaction and Fe^2+^ chelating ability (Panche et al. 2016).

### Evaluation of cholinesterase inhibitory activity

#### Evaluation of acetylcholinesterase (AChE) and butyrylcholinesterase (BuChE) inhibitory activity

**Figure 6.**
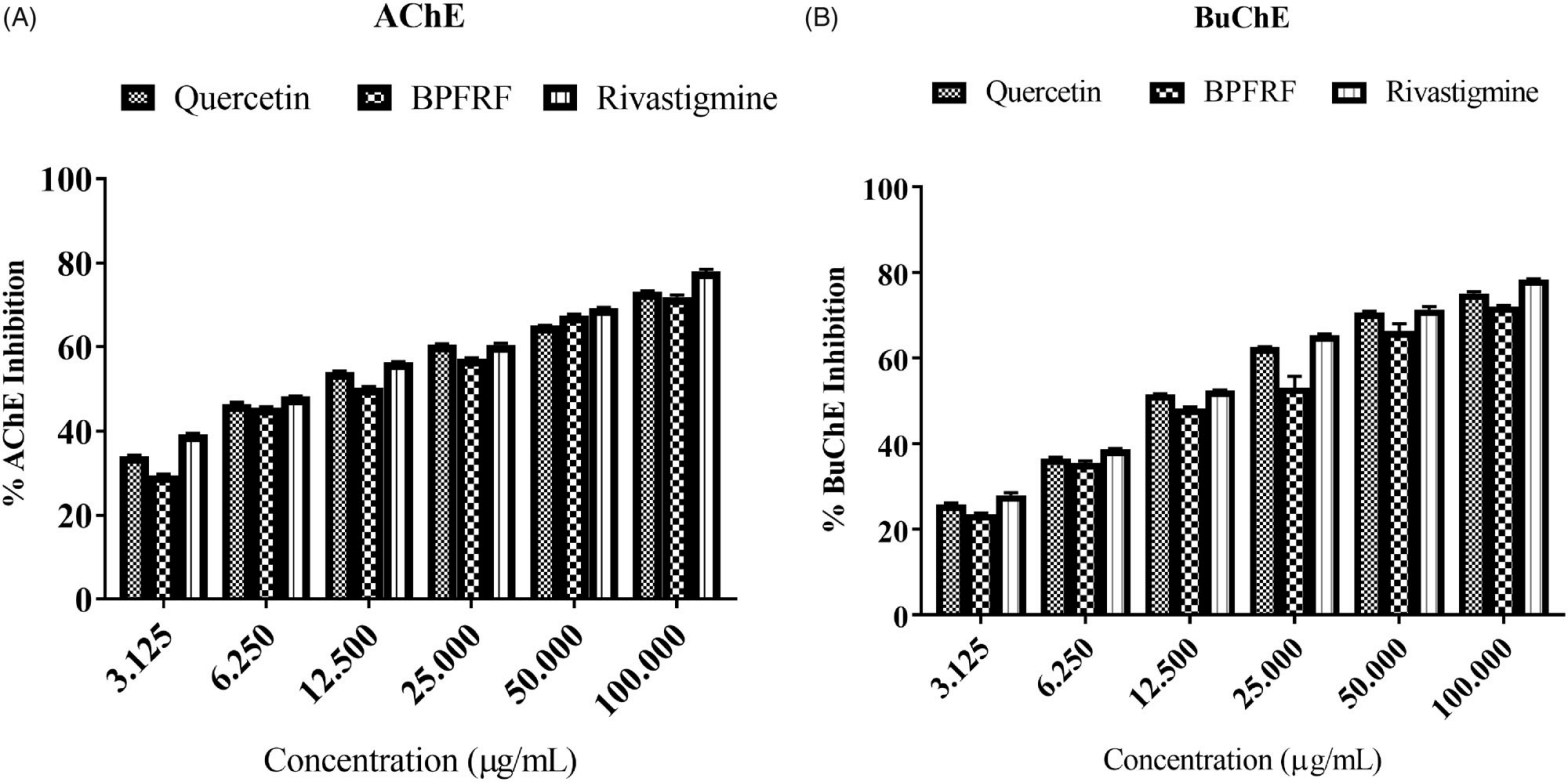
Cholinesterase inhibitory activity of BPFRF AChE (A). BuChE (B).

One of the main pathological features of AD is the decline in cholinergic acetylcholine (ACh) neurotransmitters by cholinesterases. Studies suggest that an aberrant decrease in ACh leads to cognitive dysfunction and eventually death (Sun et al. 2012). Thus, inhibition of cholinesterases is one of the central focuses on developing natural therapeutics for AD (Feitosa et al. 2011; Suganthy and Pandima 2016). The inhibitory activity of BPFRF against rat brain AChE and serum BuChE was measured. BPFRF, quercetin and the standard drug Rivastigmine exhibited a concentration-dependent increase in the inhibitory activity of AChE and BuChE, respectively Figure 6(A,B). Table 2 shows the maximum inhibitory effects of BPFRF, quercetin, and standard against AChE and BuChE. BPFRF exhibited a strong inhibitory activity, with IC_50_ values of 22.283** **±** **0.27** **µg/mL and 33.437** **±** **1.46** **µg/mL against AChE and BuChE respectively compared to quercetin (15.528** **±** **0.08** **µg/mL and 24.326** **±** **0.40** **μg/mL) and the standard Rivastigmine (7.994** **±** **0.12** **µg/mL and 20.120** **±** **0.72** **µg/mL). The IC_50_ values in µM were also presented for comparison, See Supplementary Table 1. The results obtained from this study show appreciable IC_50_ values when compared to previously reported data on different crude extracts of *B. pinnatum* (Ojo et al. 2018). This indicates that the purified BPFRF conveys a potent inhibitory effect against AChE and BuChE. Khan, Marya, et al. (2018) reported that the maximum AChE inhibitory activity of most flavonoids is due to the presence and position of the hydroxyl (OH) group at ring A and ring B and the unsaturation of ring C. The potent inhibitory activity of the partially purified BPFRF, maybe as a result of the presence of –OH groups on the side phenyl ring and interactions with important amino acid residues at the active site of the enzyme to form hydrogen bonds of the free OH groups in BPFRF flavonoids (Table 1). Thus, the anticholinesterase activity observed in this study suggests that the effect of BPFRF may not be due to only a particular flavonoid but could be due to the combined effect of the flavonoid constituents with different characteristics present in BPFRF.

### Molecular docking

A molecular docking study was performed to evaluate the binding interaction of BPFRF constituents at the active site of AChE and BuChE to gain better insight into the *in vitro* experimental results. Molecular docking studies revealed that the BPFRF constituents exhibited favourable binding energies against AChE and BuChE activity. As shown in Table 3 favourable overall binding energies were observed in ranges of −6.9 to −14.9** **kcal/mol for AChE (4EY7) −8.9 to −14.9** **kcal/mol and −12.7 to −6.9** **kcal/mol for BuChE (1POM).

**Table 3.** Binding energies of BPFRF ligands and known inhibitors against AChE and BuChE drug targets.

Compound name	PubChem ID	Chemical formula	Binding affinity (kcal/mol) AChE	Binding affinity (kcal/mol) BuChE
Luteolin-7-glucoside*	5280637	C_15_H_10_O_6_	−14.9	−9.8
Luteolin*	5280445	C_15_H_10_O_6_	−12.3	−7.4
Quercetin*	5280343	C_16_H_12_O_8_	−11.2	−7.8
Isorhamnetin*	5281654	C_9_H_8_O_3_	−10.5	−7.4
Carlinoside*	442584	C_9_H_10_O_5_	−8.9	−12.7
Rivastigmine^#^	77991	C_28_H_48_O	−10.4	−6.9

^#^Known inhibitors; *Putative BPFRF inhibitors.

The higher (negative) binding free energy, the more potent the interaction (Kumar et al. 2017). Luteolin-7-glucoside carlinoside and quercetin were found to have the highest binding affinities across the two targets compared to the synthetic inhibitors based on their binding energies (Table 3). Molecular docking provides a rough approximation of the binding affinity of a ligand for a given binding site (Ogidigo et al. 2018). The orientations of these ligands play a significant role in the interaction between active site residues (Raeisi 2013).

**Figure 7.**
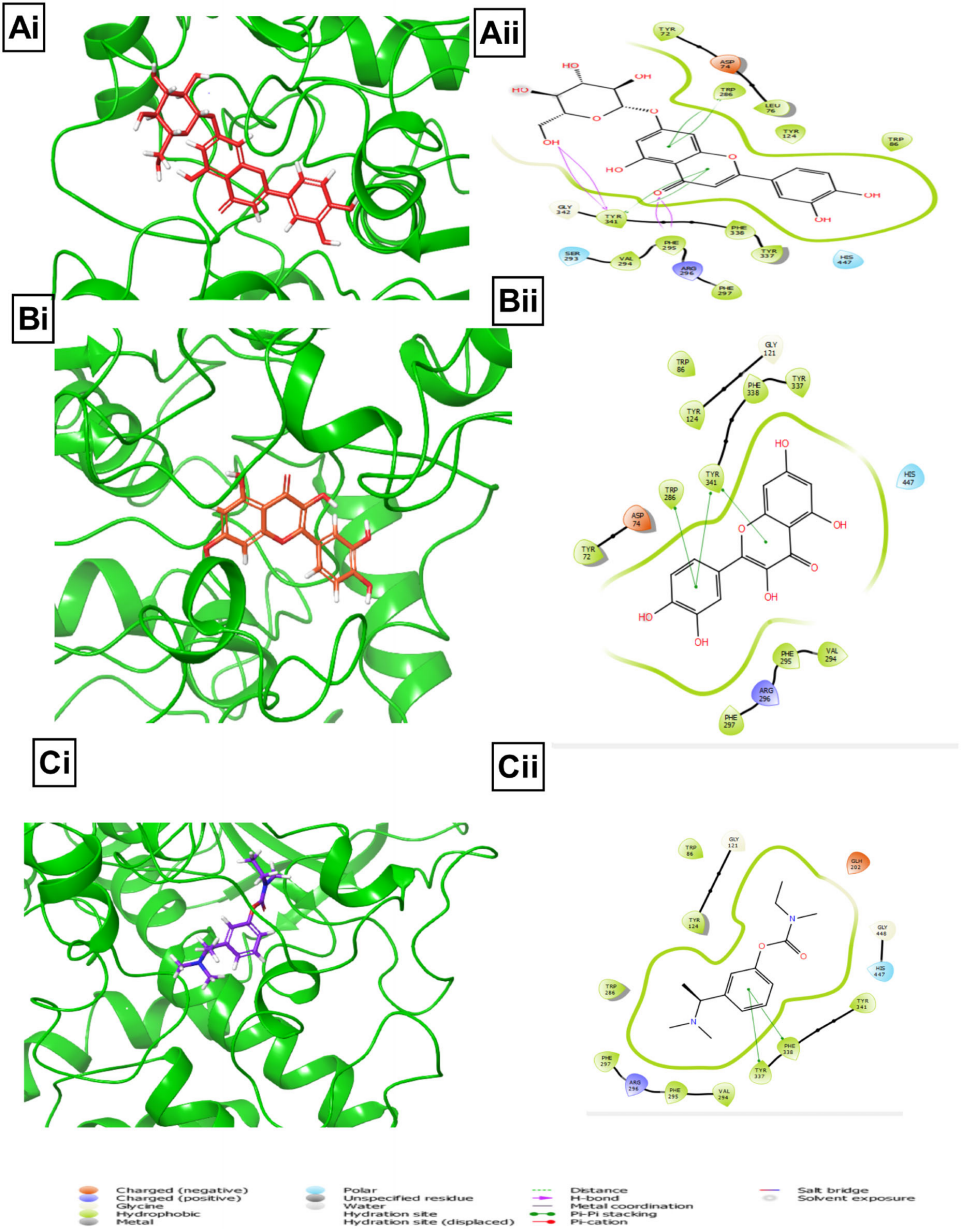
Illustrations of molecular interactions (left: 3D and right: 2D) between the/highest binding energies of BPFRF constituents and standard against AChE target. Luteolin-7-glucoside (red) (Ai, Aii). Quercetin (orange) (Bi, Bii). Rivastigmine (purple) (Ci, Cii).

In the case of AChE, the active site of the human AChE is enclosed in a 20** **Å deep pocket consisting of the catalytic site (Ser 203, His447 and Glu334), acyl-binding pocket (Phe295and Phe297) at the base of the gorge, quaternary ammonium binding locus (Trp86), oxyanion hole (Gly120, Gly121, and Ala204), and lastly, PAS (Tyr72, Asp74, Tyr124, Trp286 and Tyr341) (Johnson and Moore 2006; Kumar et al. 2017). Ligands interacting with these residues have been found to have a crucial function in the inhibition of the substrate. Figure 7(Ai,Aii) shows a 3D and 2D visual observation of the docked conformation of luteolin-7-glucoside at the AChE active site indicating interactions with the main residues at the active site. Luteolin-7-glucoside assumed a binding pose close to the catalytic anionic site (CAS) of AChE near the catalytic triad residues and formed crucial interactions. The hydroxyl group of the β-d-glucopyranosyl moiety of luteolin-7-glucoside was engaged in a hydrogen bond interaction with Tyr341, Phe295 and forming contact with an oxygen atom from the pyran ring (C-ring) Figure 7(Aii). Also, pi–pi stacking with Trp286 (PAS residue) and Tyr341 (PAS residue) was established. Polar interactions with catalytic residue His 447 was observed. The docking simulation strongly correlates well with X-ray crystallographic structures of known AChE complexes (Cheung et al. 2012; Kumar et al. 2017). Furthermore, quercetin and Rivastigmine were observed to interact with the active site residues of AChE (Supplementary Table 1 and Figure 7(B,C)).

**Figure 8.**
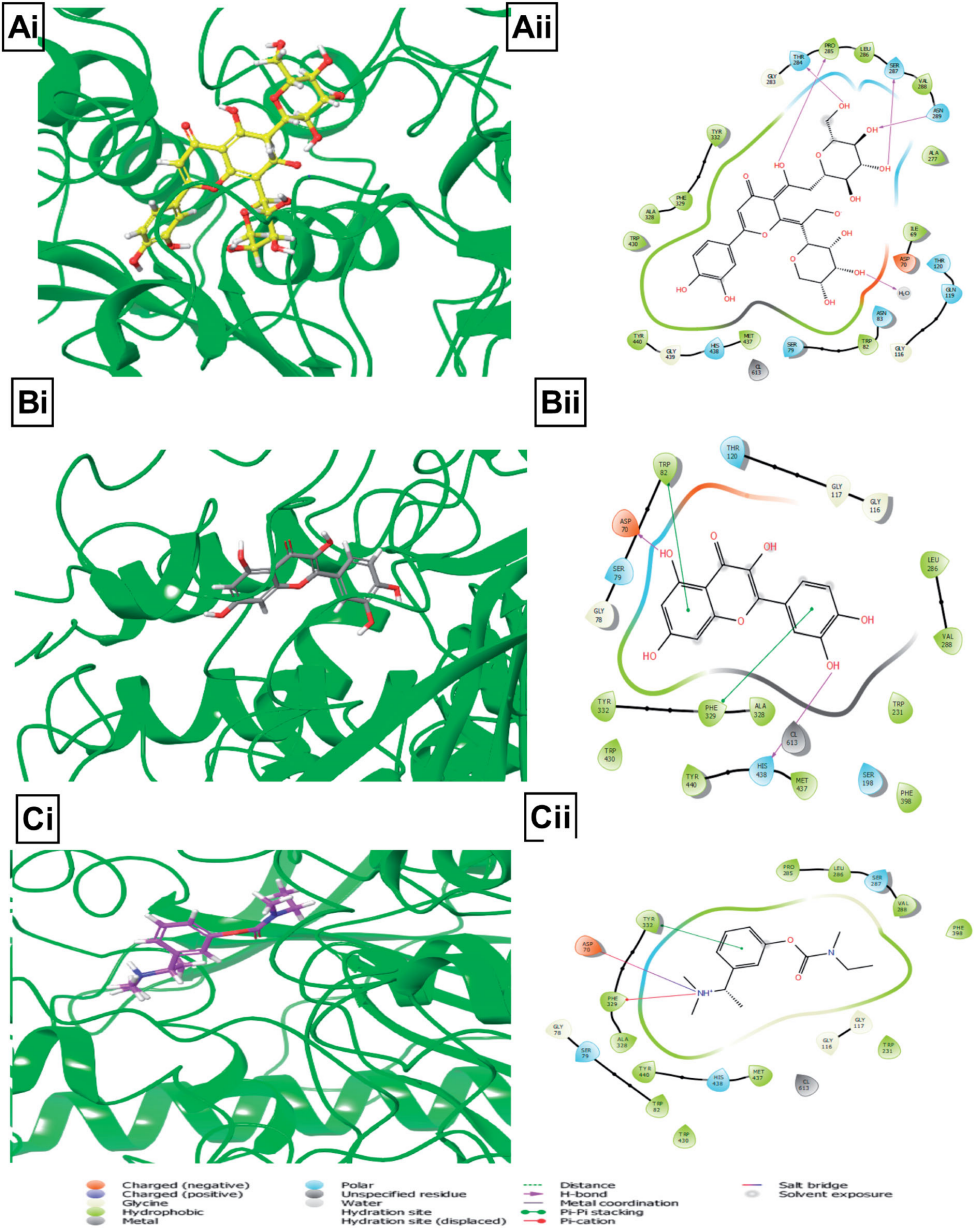
Illustrations of molecular interactions (left: 3D and right: 2D) between the highest binding energies of BPFRF constituents and standard against BuChE target. Carlinoside (yellow) (Ai, Aii). Quercetin (grey) (Bi, Bii). Rivastigmine (purple) (Ci, Cii).

For BuChE, the catalytic site of human BuChE consist of 20** **Å in its active site consisting of Ser198, His438, Glu325 catalytic amino acid residues found at the catalytic site. Other structural features of BChE include a choline-binding site or the cation-π site (Trp82), an oxyanion hole (Gly116, Gly117, Ala199), acyl binding site (hydrophobic pocket) (Leu286, Val288) and PAS (Asp70) (Kumar et al. 2017). Carlinoside showed the best binding energies with −12.7** **kcal/mol compared to the known inhibitors Rivastigmine was −6.9** **kcal/mol. Carlinoside also formed a hydrogen bond with Pro285, Ser 287, Asn 289 and noticeable polar interactions with two catalytic site residues His438 and Ser198 (Supplementary Table 2 and Figure 8(Aii)). Similarly, quercetin and Rivastigmine interacted favourably with the active site residues Ser198, His438 and Glu325 (Supplementary Table 2 and Figure 8(B,C)). These interactions observed may account for the possible mechanism of cholinesterase inhibition by BPFRF constituents. Thus, these findings fully support the *in vitro* experiment and corroborate the anticholinesterase activity of BPFRF.

## Conclusions

Profiling of flavonoids from BPFRF by UPLC-PDA-QTOF-ESIMS/MS led to the identification of 15 compounds from which one flavone derivative carlinoside was reported for the first time in this plant species. BPFRF exhibited high polyphenol content and substantial antioxidant and anticholinesterase activities. The chemical constituents of BPFRF compounds exhibited favourable cholinesterase inhibitory activities evidenced by the *in vitro* and molecular docking simulation studies. The observed biological activities of BPFRF indicate the synergistic effect of the flavonoid constituents present in *B. pinnatum* leaves. Hence, *B. pinnatum* could emerge as an effective cholinesterase inhibitor and potent antioxidant agent against oxidative stress and free radical damage associated with AD; thus, these results provide a basis for the clinical validation of *B. pinnatum* as a promising source of neuroprotectant.

## Supplementary Material

Supplementary_Data_PHB.doc

## Data Availability

The data that support the findings of this study are available from the authors upon request.
