# Supplementary material for: UPLC-PDA-ESI-QTOF-MS/MS fingerprint of purified flavonoid enriched fraction of Bryophyllum pinnatum; antioxidant properties, anticholinesterase activity and in silico studies
Source: Pharm Biol. 2021 Apr 30;59(1):444–56. doi: 10.1080/13880209.2021.1913189 (PMC8871626; doi:10.1080/13880209.2021.1913189)
Supplement: Supplementary_Data_PHB.doc [file IPHB_A_1913189_SM1953.doc]

Supplementary Data

Supplementary Table S1: DPPH radical scavenging potential, lipid peroxidation inhibitory activity and cholinesterase inhibitory activity of BPFRF and standards.

Supplementary Table S1: Molecular interaction of AChE and BuChE active sites with constituents of BPFRF and known inhibitors

Table S1: DPPH radical scavenging potential, lipid peroxidation inhibitory activity and cholinesterase inhibitory activity of BPFRF and standards.

| **Samples** | **DPPH**  (µM) | **LP**  (µM) | **AChE**  (µM) | **BuChE**  (µM) |
| --- | --- | --- | --- | --- |
| BPFRF | - | - | - | - |
| Ascorbic Acid | 57.83 ± 0.827 | 81.72 ± 0.309 | - | - |
| Quercetin | 45.66 ± 1.125 | 83.51 ± 0.654 | 51.39 ± 0.289 | 97.17 ± 1.324 |
| Rivastigamine | - | - | 31.93 ± 0.409 | 80.37 ± 2.905 |

**Table S2**: Molecular interaction of AChE and BuChE active sites with constituents of BPFRF and known inhibitors.

|  | | | **BPFRF constituents** | | **Major interactions** | **Active site residues within 4 Å of the docked ligand** |
| --- | --- | --- | --- | --- | --- | --- |
| **AChE** | | | | | | |
|  | | Luteolin-7-glucoside | | | H- BOND : TYR341;  PI-PI STACKING : TRP: 286, TYR 341 | TYR 72, ASP 74, LEU 76, TRP 86, TYR 124, TRP 286, SER 293, PHE295, ARG 296, PHE 297, PHE 338, TYR 341, HIS 447, |
|  | | Luteolin | | | H-BOND: TYR 72 , ASP 74, PHE 295 ;  PI-PI STACKING: TYR 34 , TRP 286, | TYR 34, TYR72, ASP 74, TRP 86, TYR124, TRP286, VAL 294, SER 293, PHE 295, ARG 296, PHE 297, HIS 447 , |
|  | | Rivastigmine | | | PI-CATION: TYR 124 | TYR 72, ASP 74, TYR 124, TRP 286, VAL 294, PHE 295, ARG 296, TYR 337, PHE 338, TYR 341, HIS 447 |
| **BuChE** | | | | | | |
|  | Carlinoside | | | H- BOND : ASN 289, SER 287, THR 284, PRO 285 | | GLY 11, THR 20, SER 79, ASN 83,TRP 92, GLN 119, THR 284, PRO 285, LEU 286, SER 287, ASN 289, VAL 288, ALA328, PHE 329, TYR 332, HIS 438, TYR 440 |
|  | Luteolin-7-glucoside | | | H- BOND : LYS 85,  ASN 186, VAL 135, | | THR 20, SER 79, ASN 83, TRP 92,GLY 117, GLN 119, THR 120, SER 198, TRP 231, LEU 286, VAL 288, TRP 430, MET 437, HIS 438, GLY 439, TYR 440 |
|  | Quercetin  Rivastigmine | | | H-BOND: TRP 82, HIS  438; PI- PI: STACKING: PHE 329  H-BOND: SER 287 | | PHE 39,ASP 70, SER 79,TRP 82, GLY 116, GLY 117, SER 198, GLN 119, THR 120, TRP 231, PRO 285, LEU 286, SER 287, VAL 288, ALA 328, PHE 329, TRP 430, MET 437, HIS 438, TYR 440, GLY 439,    PHE 39, ASP 70, SER 79, TRP 82, GLY 116, GLY 117, GLN 119, SER 198, THR 120, TRP 231, LEU 286, VAL 288, PRO 285, SER 287, ALA 328, PHE 329, TYR 332, TRP 430, MET 437, HIS 438, GLY 439, TYR 440 |
